# Agriculture in Africa – Telling myths from facts: A synthesis^☆^

**DOI:** 10.1016/j.foodpol.2017.02.002

**Published:** 2017-02

**Authors:** Luc Christiaensen

**Affiliations:** Jobs Group, World Bank Group, USA

**Keywords:** Agriculture, Africa, Measurement, Technology, Structural transformation, Market

## Abstract

Stylized facts drive research agendas and policy debates. Yet robust stylized facts are hard to come by, and when available, often outdated. The 12 papers in this Special Issue revisit conventional wisdom on African agriculture and its farmers’ livelihoods using nationally representative surveys from the Living Standards Measurement Study-Integrated Surveys on Agriculture Initiative in six African countries. At times they simply confirm our common understanding of the topic. But they also throw up a number of surprises, redirecting policy debates while fine-tuning others. Overall, the project calls for more attention to checking and updating our common wisdom. This requires nationally representative data, and sufficient incentives among researchers and policymakers alike. Without well-grounded stylized facts, they can easily be profoundly misguided.

## 1 Introduction

Stylized facts drive research agendas and policy debates. They provide a sense of importance, help frame the inquiry and are used to galvanize resources. So, the notion that 60–80 percent of work in African agriculture is done by women has often been quoted to motivate a greater gender focus in agricultural research and policymaking. Similarly, the observation that one third of the world’s food is lost post-harvest, is used to rally the world around a food waste reduction agenda (Chaboud and Daviron, 2017).

Yet, robust stylized facts, systematically obtained with reliable methodologies and comparable data across countries, and settings within countries, are often hard to come by. Partly this is because some concepts are simply difficult to measure. Not everything important can come packaged as a neat statistic. In the face of pressure to produce statistics irrespectively, wrong-headed numbers may arise. It partly also reflects the lack of regular, representative and reliable data to compile these facts. Finally, preoccupation with causal inference often leaves few incentives to produce a ground-truthed description of reality. With stylized facts often constituting the very starting point of research itself, this is odd at best.

As a result, academic debates and policies rely too often on outdated or poor quality statistics, or just unrepresentative case study evidence. In Jerven’s words (2016, p. 343): “*… the numerical basis on which we study African economies is poorer than we would like to think.*” Sometimes numbers have even evolved into zombie statistics, numbers that live a life on their own, with their empirical basis undocumented and their origins unknown, though widely accepted as conventional wisdom, such as the notion that 70 percent of the world’s extreme poor are women.^1^ Devarajan (2013) calls for urgent action to remedy such “statistical tragedy”. After all, research, policies and investments can only be as good and effective as the data and evidence informing them (Beegle et al., 2016, Jerven, 2016).

The topic of quality data and measurement has recently started to receive more attention, in the literature and in policy circles, especially for macroeconomic statistics (Jerven, 2013), literacy (UNESCO, 2015) and poverty (Beegle et al., 2016). But the need to revisit common wisdom applies equally to agriculture, and in particular, African agriculture (Carletto et al., 2015a). The world in which African agriculture operates has been changing dramatically over the past two decades, following robust economic growth, rapid urbanization, and climate change. But the information base on African agriculture has been limited for a long time, often even lacking reliable statistics on basic metrics such as the country’s agricultural yields. More generally, translating economic concepts into numbers, such as the notions of productivity, seasonality, commercialization, or a households’ net food marketing position (net buyer/seller), remains intrinsically challenging,^2^ often requiring special data that are not standardly collected at scale, forcing analysts to rely on outdated or case study evidence or proxy measures instead.

The household survey panel data collected under the Living Standards Measurement Study–Integrated Surveys on Agriculture (LSMS-ISA) Initiative provide a unique opportunity to take up this challenge. Over the period 2008–2020, nationally representative surveys are to be conducted in 8 African countries, representing 45 percent of Sub-Saharan Africa’s (SSA) population. In these countries, four or more waves of detailed information are collected on households’ economic activities, their income and well-being, with special attention to agriculture. They also include a number of methodological innovations such as data gathering at the individual and plot level, enabling more gender disaggregated analysis. The data are made publicly available one year after their collection.^3^

An international consortium of researchers under the Agriculture in Africa – Telling Myths from Facts project led by the World Bank, with complementary financing from the African Development Bank,^4^ exploited the first rounds of these surveys to revisit common wisdom on African agriculture and its farmers’ livelihoods in the areas of agricultural technology, market engagement and structural transformation. Studies were each time framed around a cross-country investigation of conventional wisdom in these areas. A total of 12 broad stylized facts and sub-facts on African agriculture and rural livelihoods were thus reviewed, the results of which are brought together in this special issue.

This synthesis summarizes the key findings, including through a number of easily accessible and replicable tables and figures, and reflects on their implications. It seeks to facilitate the policy dialogue and further research efforts including updating as new information from LSMS-ISA or related surveys becomes available. The findings at times confirm conventional knowledge, as one would hope, and put it on more solid empirical footing. More often they fine-tune our understanding. But they also reveal some myths and raise new issues. Overall, the findings underscore the high academic and policy return from investing in regular, nationally representative data collection and continuous examination of conventional wisdoms.

The synthesis proceeds as follows. The next section expands on the underlying data base and the methodological approach taken. This precedes a synoptic overview of the 12 wisdoms revisited and the core findings obtained when submitting them to the data. Section three expands on each of them, including their implications for agricultural and rural development policies. Section four concludes.

## 2 Myths, materials, and methods

The LSMS-ISA Initiative^5^ supports national statistical offices in the collection of at least four rounds of nationally representative household panel survey data in eight African countries during 2008–20. The papers in this study mainly draw on the first rounds collected during 2009–2012 in 6 of these countries (Ethiopia, Malawi, Niger, Nigeria, Tanzania and Uganda). They cover more than 40 percent of the population in SSA and most of its agro-ecological zones. While this does not make them representative for SSA as such, together they provide a broad picture of the emerging new reality, and also allow for elucidating differences across settings. In these countries, a total of 31,848 households were interviewed, with sample sizes per country varying between 2716 (Uganda) and 12,271 households (Malawi), of which, on average, 76% were rural. Burkina Faso and Mali have joined the Initiative more recently. Their survey findings are not included here.

The LSMS-ISA initiative also presents a number of notable innovations on the World Bank’s Living Standards Measurement Study (LSMS) surveys, which for some time provided important information on the lives of Africans, their income, their economic activities, and their wellbeing. Most importantly, it strengthens the coverage of household agricultural activities—the Integrated-Surveys-on-Agriculture part of LSMS-ISA. The surveys are based on household samples and designed from the perspective of the household, not the farm. As a result, medium and large scale farms are only sparsely covered in practice (Jayne et al., 2016), even though technically represented in the sample. Information is gathered at both the household and the plot level, covering every aspect of farmers' life—from the plots they cultivate, the inputs they use, the crops they grow, the time they allocate per plot, the harvest that is achieved, the way they market it, the amount they lose post-harvest, and so on.

Second, in addition to the integrated approach to data collection, data gathering takes place at highly disaggregated levels, at the plot level, but also at the individual level, such as for time allocation and plot management. This enables a more refined, gendered perspective on agriculture and rural livelihoods. Third, the surveys make wide use of ICT-tools. Tablets are used for data collection, improving the quality of data (Caeyers et al., 2012); households are geo-referenced using Global Positioning System (GPS) devices, enabling further integration with other data sources, and plot size is measured by GPS as opposed to self-reporting, improving accuracy of land based statistics (Carletto et al., 2015b). Finally, individuals (not just households) are tracked across survey rounds, opening a host of new research areas such as the study of migration.

These four innovative features of the data (integration, individualization, ICT use and intertemporal tracking) not only help obtain a more refined insight in African agriculture and its rural livelihoods, they also help scrutinize conventional views that have so far lacked an adequate information base to do so, such as the gender patterns in agricultural labor allocation or the application of joint input packages in practice, i.e. at the plot level. The nationally representative scope of the data and the great degree of standardization across countries in questionnaire design and survey implementation further facilitate cross-country comparison as well as comparisons across settings within countries.

Given the core objective of establishing solid statistics and distinguishing myths from facts, the studies have been primarily descriptive in orientation, focusing on a careful definition and empirical operationalization of the concepts at hand. Regression analysis is mainly used to complement the findings, to check robustness and generate hypotheses, not for causal inference. For this reason, the panel nature of the data has not been exploited much here, and the focus has been limited to the first rounds of the data. Cross-country comparisons were systematically undertaken, though only when data comparability permitted it to do so.

The twelve topics examined were chosen following a review of key policy documents and expert consultations, and because of their salient nature in ongoing academic and policy debates, in addition to the feasibility of the new LSMS-ISA data to address and advance these debates. The papers speak to the prevailing, overarching notions that (1) Africa’s agricultural technology is backward; (2) that smallholder engagement with input, factor and product markets remains limited and (3) that Africa and its citizens are behind in the structural transformation of their economies, occupations and incomes. Obviously, the papers address only a small subset of the stylized facts related to these and other topics on African rural development. Nonetheless, the facts revisited have been driving a number of the contemporaneous debates on African agriculture and in initiating this endeavour, the project also seeks to catalyze a process of continuous fact-checking moving forward.^6^

Table 1 provides a schematic summary of whether the conventional views reviewed in these areas do indeed stand the test of the data, and to what degree. Are they myths in today’s African farming context, or realities?

But the answer to the ‘Myth versus fact?’ question is certainly more complex than suggested in this table. Farming and farming behavior are complex, and the concepts and statistics we use to describe it, are unlikely to be as cooperative as the table suggests. Reality also varies—across farming systems, regions and countries, and over time. The studies reflect this complexity, and explore the nuances that any answer to the question ‘myth versus fact?’ has to exude.

## 3 A micro-economic update on African agriculture

### 3.1 Backward technology

The prevailing view about African agriculture is that technology is backward, and changing only slowly. Africa is decades behind Asia from this perspective. Farmers are slow to respond to modern methods of farming such as the use of modern inputs and mechanization, land improvement and irrigation. Official estimates put cereal yields in SSA still only at about 1.5 ton/ha on average (2012–2014), about half those in South Asia (3.1 ton/ha) and a quarter those in China (5.9 ton/ha) (World Bank, 2017).

This is a major concern. Two in five Africans still live in extreme poverty (Beegle et al., 2016) and boosting agricultural productivity is key for poverty reduction (Christiaensen et al., 2011). It is also somewhat surprising as the Boserup-Ruthenberg (BR) theory of agricultural intensification (Boserup, 1965, Ruthenberg, 1980) would have predicted higher uptake and the technologies are generally believed to be profitable (Byerlee et al., 2007). Substantial gender gaps in agricultural productivity (O’Sullivan et al., 2014), combined with the notion that African women perform most of the work in agriculture further suggests that shifting attention to female farmers to close this gap could be an important avenue to boost agricultural output.

The first four papers of this special issue confront these conventional wisdoms with the data. They begin with an update of the extent of input use (Sheahan and Barrett, 2017). Binswanger-Mkhize and Savastano (2017) then assess the current input application rates within the macro-context of Africa’s current population density and market access. This is followed by a case study of the actual profitability of fertilizer use in Nigeria (Liverpool-Tasie et al. (2017)), drawing attention to a core micro-economic principle driving input adoption, namely profitability. The fourth paper explores the potential for increasing crop output from closing the gender productivity gap (Palacios-Lopez et al., 2017b).

**Fig. 1:**
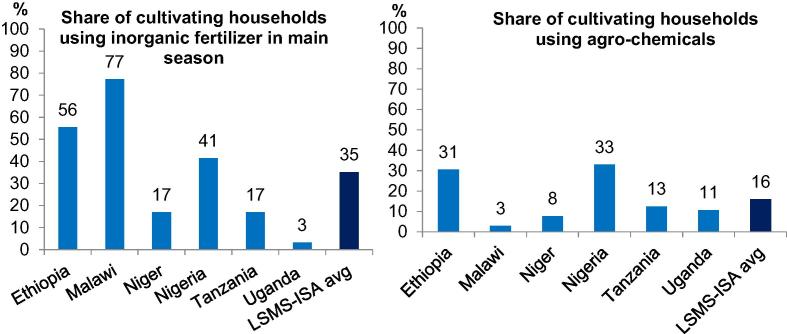
Modern input use in SSA is not uniformly low. **Note**: Agro-chemicals include pesticides, herbicides and fungicides.

Source: Sheahan and Barrett (2017).

***African farmers do in fact use modern inputs, even though not always efficiently.*** According to common wisdom, farmers in Africa hardly use modern inputs such as inorganic fertilizer and other agro-chemicals, or mechanization and water control. Using data from over 22,000 households and 62,000 agricultural plots from the six LSMS-ISA countries, Sheahan and Barrett (2017) revisit this record, offering a number of “potentially surprising” facts. They find that fertilizer and agro-chemical use is more widespread than is often acknowledged. One third of the cultivating households in the LSMS-ISA countries apply inorganic fertilizer and the average unconditional nutrient application rate is 26 kg/ha (corresponding to 57 kg of total fertilizer/ha). This is twice the SSA average of 13 kg of nutrients/ha during the same period, even though still only one fifth of the OECD average.^7^ But rates vary considerably across countries (and also across regions within countries). Use is highest in Malawi, Ethiopia and Nigeria, where more than 40 percent of cultivating households apply inorganic fertilizer, but much lower in Niger and Tanzania (17%) and Uganda, where inorganic fertilizer use is virtually nonexistent (Fig. 1).

One in six farmers also uses agro-chemicals, rising to one in three in Nigeria and Ethiopia—related to the more widespread use of herbicides in these countries.^8^ These rates are substantially higher than the available numbers in the literature, and come somewhat as a surprise. It prompted follow up work, also using the LSMS-ISA data, which suggests a strong correlation of pesticide use with increased value of the harvest, but also with increased health expenditures and time lost from work due to sickness. This draws attention to the health implications of increased agro-chemical use in SSA and the need for effective regulatory policies as areas for future attention (Sheahan et al., 2016). There are also signs of improved seed use, especially for maize, with 24 to 41 percent of maize growers reporting seed purchases, though these are likely lower bounds.^9^ Input use appears also no longer confined to traditional cash crops, with input intensification (fertilizer, pesticides, improved varieties) now equally (and at times even more) common for the maize staple.

**Fig. 2:**
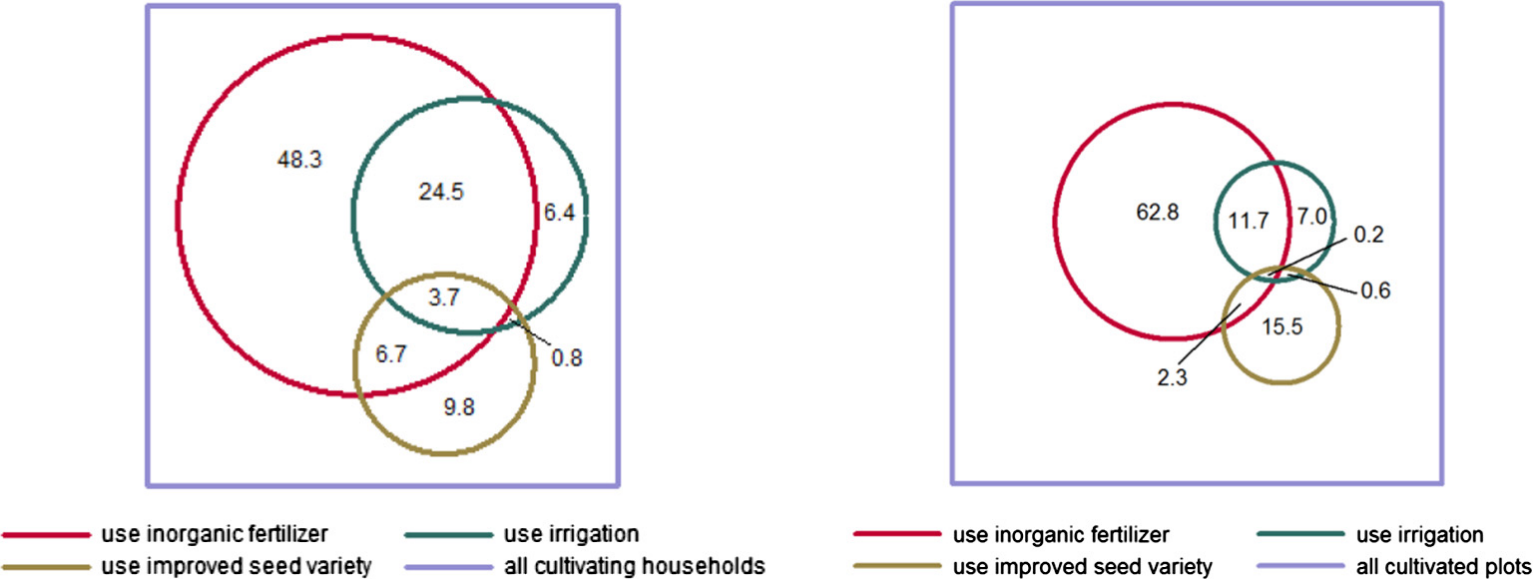
Synergies from joint input use are essentially foregone. **Note**: The areas of the circles proportionally represent population size relative to the full sample of cultivating households. The percentages in the circles are conditional on using any one of the three included inputs (i.e. exclude the population that does not use any of the three inputs) and are not weighted.

Source: Sheahan and Barrett (2017).

But it’s not all good news. With only 32 percent of cultivating households in the LSMS-ISA countries owning and 12 percent renting some type of farm equipment (less than 1 percent own a tractor) and less than 5 percent using some form of water control (2 percent of the cultivated area), the incidence of mechanization and irrigation remains quite small. Farmers also fail to benefit from the synergetic use of inputs, using them mainly as substitutes instead of complements.^10^ For example, only about a third of households in Ethiopia who apply at least one of three synergetic inputs (inorganic fertilizer, improved seeds and irrigation) apply two or more. This reduces to only 15 percent when plots are considered (Fig. 2). It points to the lack of agronomic knowledge or perhaps the underestimated complexity of joint input application, which under certain circumstances might make it rational to use them as substitutes rather than as complements. Perhaps the biggest message of the study though is that the country setting is the main factor behind farmer input use—the policy and market environments really do matter.

***Agriculture intensification remains below what increased population pressure and market access would suggest.*** Sheahan and Barrett (2017) highlight that the use of modern inputs is no longer universally low, especially for inorganic fertilizer and maize, a key staple. But in their paper, Binswanger-Mkhize and Savastano (2017) are less sanguine about the current state of affairs. In light of Africa’s increased population pressure and market access, they argue that higher degrees of agricultural intensification should be observed. That’s at least what the longstanding Boserup-Ruthenberg farming systems theory of agricultural intensification would suggest. In this view, a virtuous circle of intensification emerges, whereby population growth and market access reduce the length of fallow and induce the higher use of organic manure and fertilizers to offset declining soil fertility, as well as investments in irrigation and mechanization. Together these offset the negative impact of population growth on farm sizes, maintaining or increasing per capita food production and farm income. Alternatively, if the intensification triggered by population growth and market access is insufficient to raise food production and farmers’ incomes, beyond those of their parents, agricultural involution would be observed instead (Geertz, 1963).

**Fig. 3:**
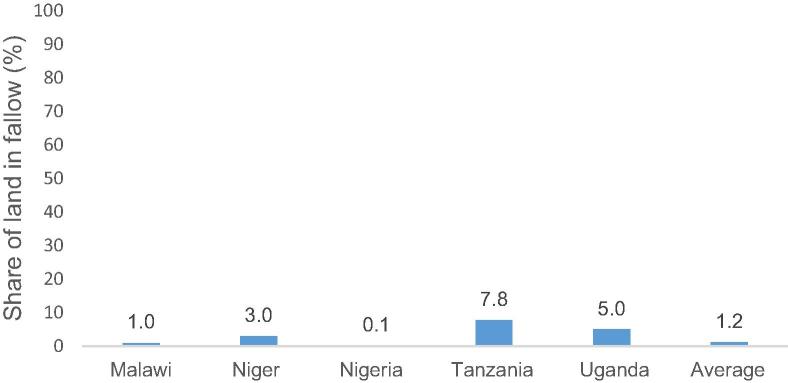
Fallow land has virtually disappeared, except in Tanzania.

Source: Binswanger-Mkhize and Savastano (2017).

The authors set out to explore the relevance of the BR framework in understanding contemporaneous modern input use in SSA (acknowledging that longer term panel data would be needed for proper testing). They find only partial support. They establish that fallow areas have virtually disappeared (on average the rate of fallow in the six countries is 1.2 percent (Fig. 3)), an important finding that has not been systematically documented so far. They also observe a response of modern input use (fertilizer, agro-chemicals and improved seeds) to their newly developed exogenous measure of agro-ecological potential, which is correlated with population density, and controlling for market accessibility (as measured through their newly developed measure of urban gravity), but not with other measures of intensification, such as irrigation or crop intensity. Overall, they conclude that the existing use of organic and inorganic fertilizer is insufficient to maintain soil fertility when fallow practices cease. And the weak response of crop intensity and irrigation is also not consistent with the BR framework. In light of the promising outcomes suggested by the BR framework, the process of intensification across these countries appears to have been too weak according to the authors.

The conclusion by Binswanger-Mkhize and Savastano is consistent with the prevailing notion of underutilization of modern inputs in Sub Saharan Africa. But it remains unclear what an acceptable level of intensification should be, given Africa’s current state of population pressure and domestic market access. Intensification is clearly starting to happen in some of the more densely populated landlocked countries and areas within these countries, and has also been accompanied by a decline in poverty, as in Ethiopia. Globalization and Africa’s resource boom of the past two decades have further enabled governments and farmers to meet food needs through an expansion of food imports and rural-urban migration (as in Nigeria), which may also have raised the levels at which population pressure really starts to bite and governments start to act upon it.^11^ So is the glass half full with respect to Africa’s agricultural intensification, as the findings by Sheahan and Barrett (2017), using the same the data, would suggest, or is it half empty, as Binswanger-Mkhize and Savastano (2017) would hold?

***Returns to fertilizer use are not always favorable—at least in Nigeria*:** Another, more direct way to assess whether modern inputs are underutilized, is to examine their profitability. The notion that fertilizer use is too low is predicated on the assumption that it is profitable to use fertilizer at higher rates than currently observed (Byerlee et al., 2007). There is, however, surprisingly limited empirical evidence to support this. Liverpool-Tasie et al. (2017) examine the profitability of fertilizer use in maize production in Nigeria, where fertilizer use is already relatively high. Production theory suggests two criteria to assess profitability of input use. The first (and weaker) criterion holds that fertilizer use is profitable when the overall net return is positive, i.e. as long as the value of the *average* kg of maize produced per kg of fertilizer (i.e. the average value product, AVP) is higher than the price per kg of fertilizer; the average value cost ratio (AVCR) is greater or equal than one. The second (and more widely used) criterion holds that fertilizer use is profitable, when it is optimal or profit maximizing, i.e. as long as the value of the *additional* maize produced per kg of fertilizer (i.e. the marginal value product, MVP) equals the price of fertilizer; the marginal value cost ratio (MVCR) equals one.

Application of these criteria to maize producers in the cereal-root crop farming system^12^ in Nigeria suggests that current application rates yield a negative return for almost half the plots (AVCR < 1) and that only about half the plots would gain from expanding fertilizer use (MVCR > 1). AVP and MVP estimates are derived from plot level regressions with household fixed effects augmented with several time invariant plot characteristics. The findings are partly due to the high transport costs involved in procuring fertilizers from the nearest distribution points (Table 2). Setting these so-called “last-mile(s)” procurement costs to zero, as if the fertilizer were directly available on the farm, would raise the number of plots where fertilizer use pays (AVCR > 1) to 85–90 percent, and increase the percentage of plots that could gain from adding fertilizer to over 70 percent. The importance of the “last mile(s)” for fertilizer acquisition costs has also been raised by Minten et al. (2013) who report that farmers in Ethiopia living about 10 km away from a distribution center faced transaction and transportation costs (per unit) that were as large as the costs to bring fertilizer over approximately a 1000 km distance from the international port to the input distribution center.

But the limited profitability of fertilizer use in the Nigerian sample is also due to poor marginal yield responses. At an average of about 7.7 kg additional maize per additional kg nitrogen, these are well below the marginal physical products observed in other studies (ranging between 10 and 20 in Kenya) or when research management protocols are followed (rising to 50 in Malawi) (Marenya and Barrett, 2009, Matsumoto and Yamano, 2011, Sheahan et al., 2013, Snapp et al., 2014). In this context, Marenya and Barrett (2009) also point to the importance of good quality soils for inorganic fertilizer to be effective. The efficiency of inorganic fertilizer is for example low on soil with low organic matter content which is needed to prevent run-off and leaching and for gradual nutrient release. Efficient absorption of nutrients is similarly impeded when soil is too acidic. Both are common problems of African soils (Barrett et al., 2017).

While the relatively high inorganic fertilizer application rates observed in Nigeria are exceptional across the LSMS-ISA countries (Sheahan and Barrett, 2017), the findings by Liverpool-Tasie et al. (2017), which, in Nigeria, are also confirmed for rice (Liverpool-Tasie, 2016), underscore the need to better understand the agro-ecological and market conditions under which inorganic fertilizer use in particular, and other agricultural technologies in general, are profitable. Also, in the absence of adequate ex post coping mechanisms, still higher returns will be needed for fertilizer (and other modern) input use to be profitable or optimal (Dercon and Christiaensen, 2011). The results reported here have abstracted from risk considerations. They also underscore the importance of integrated interventions (access to input use, extension, and markets).

***Women do not provide the bulk of labor in African agriculture.*** There is also a gender dimension to low modern input use, with application rates typically lower among female headed households and on female managed plots (Sheahan and Barrett, 2017). This explains an important share of the 20–25 percent gender gap in agricultural productivity (O’Sullivan et al., 2014). Combined with the widely accepted notion that women provide the bulk of the labor in agriculture in Africa, regularly quoted to be 60–80 percent, this has been taken to suggest that closing the gender productivity gap could go a long way in boosting Africa’s food supply. But the basis for this statistic on women’s labor share in Africa’s agriculture is basically unknown and has been questioned (Doss et al., 2011).

**Fig. 4:**
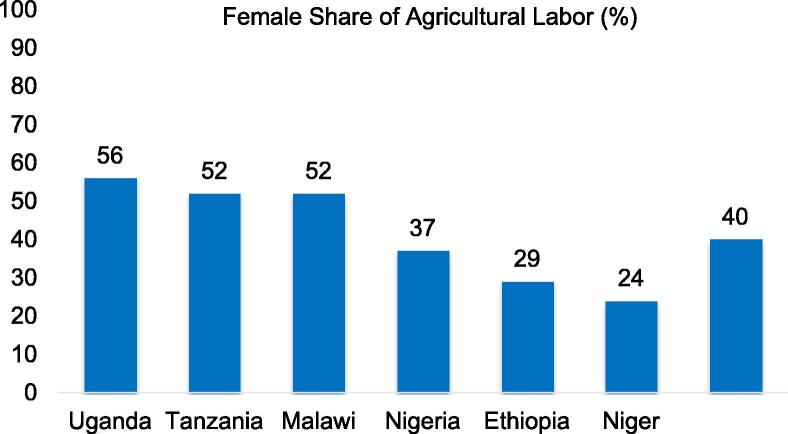
Women do not provide the bulk of labor in African agriculture. **Note**: ∗ Population weighted.

Source: Palacios-Lopez et al. (2017).

Exploiting the plot level labor input records for each household member across the six LSMS-ISA countries, Palacios-Lopez et al. (2017b) find that women contribute just 40 percent of labor input to crop production. The numbers are slightly above 50 percent in Malawi, Tanzania and Uganda, but substantially lower in Nigeria (37 percent), Ethiopia (29 percent) and Niger (24 percent) (Fig. 4). The difference in the contribution in Nigeria between the northern (32 percent) and southern (51 percent) regions is illustrative and tallies with expectations. It confirms heterogeneity across and even within countries. Controlling for the gender and knowledge profile of the respondents does not meaningfully change the predicted female labor shares. Across the different countries, there are also no systematic differences across crops or activities.

The authors conclude that the female labor share statistics in Africa’s agriculture do not, as such, support a (universally) disproportionate focus on female farmers to boost crop production. They further highlight the need to use consistent metrics when analyzing the benefits and costs of different interventions, as the gender productivity gaps are in fact calculated based on differences in land productivity among male and female managed plots, as opposed to differences in returns to labor. The agricultural labor shares are in fact irrelevant for such calculations, irrespective of their size. That said, there may be many other important reasons for investing in raising female labor productivity in agriculture, such as female empowerment and improving the nutritional outcomes of children. Establishing this requires further research for which nationally representative and gender disaggregated household survey data on time use and intra-household control of income and productive resources will be key. The new LSMS-ISA survey rounds make important steps in this direction, creating promising opportunities for future research on gender and agriculture in Sub-Saharan Africa.

### 3.2 Poor market functioning

A second recurring theme in the academic and policy debates on African agriculture and rural development is the poor functioning of input, factor and product markets (Barrett et al., 2017). Land, labor and credit markets are considered largely absent, even 20 years after the structural adjustment era of the 1980–1990s, impeding modernization and commercialization of agriculture. Most recently, it has among others given rise to the (re)-introduction of fertilizer programs (see Jayne et al., 2013 for an assessment). The lack of smallholder market participation is considered to be holding back progress in the fight against malnutrition (von Braun and Kennedy, 1994), with 38 percent of African children under the age of five still suffering from growth retardation (Beegle et al., 2016). A related manifestation of market failures is the presence of food price seasonality, a widely acknowledged, but little systematically documented and increasingly neglected phenomenon (Devereux et al., 2011).

The following five papers in the special issue query the prevailing notions of continuing factor market imperfection (Dillon and Barrett, 2017, Deininger et al., 2017, Adjognon et al., 2017), limited commercialization and its effect on nutrition (Carletto et al., 2017), and food price seasonality (Gilbert et al., 2017).

***Factor markets in general don’t function well****.* The conventional wisdom sees African agriculture trading in missing or imperfectly functioning factor markets. Dillon and Barrett (2017) conclude that this is largely true. At the heart of this finding is the simple observation that the number of working age people in the household should not affect the amount of labor used on the farm if factor markets function well. If the size of the household does affect the amount of labor used on the farm, clearly factor markets (not only labor markets) are either absent or functioning poorly. This test goes back to Benjamin (1992) and has been applied in a number of settings (Udry, 1999, LaFave and Thomas, 2014). The authors apply it systematically across five LSMS-ISA countries.

They find a significant link between labor input and household size, across all countries. The link is further robust to the gender of the household head, the distance from markets and the agro-climatic environment, suggesting that rural factor market failure is pervasive and structural. Yet, they also find that rural factor markets are not generally missing in an absolute sense. On average across countries, 29.4 percent of agricultural households rent/borrow land, 38.9 percent hire labor and 23.7 percent take out a loan (Table 3). Market existence thus appears less of a problem than market function. Further work is needed to unpack the sources (e.g. labor, land or financial markets) and causes of the underlying market failures to help target the necessary interventions.

***But land markets already perform a useful role:*** Deininger et al. (2017) explore in greater depth and more directly, the extent to which farmers are engaged in land markets, and the nature of that engagement. They confirm that farming households are already more actively engaged in land markets than commonly assumed, especially in land rental markets (Table 3). Land sales activity remains limited, though information was only available for Niger and Uganda.

Rental market access proves to have significant and beneficial effects for the equalization of land endowments and farm productivity. It permits land-poor but labor-rich households to raise their resource base by renting in land. It facilitates other farmers to diversify their activity by renting out their land and taking up non-farm employment (without the risk of losing their land assets). These are profound gains in a process of structural change. The effects are strongest in Malawi, Nigeria and Uganda.

The authors suggest that institutional reforms (especially within the legal framework) are needed and effective in strengthening the role that land markets play in enhancing farmer welfare. They especially call for a more differentiated and empirically grounded view of the reality farmers face on land, which should be combined with a thorough understanding of the institutional context. They make a number of suggestions on how household questionnaires could be improved to achieve this.

**Fig. 5:**
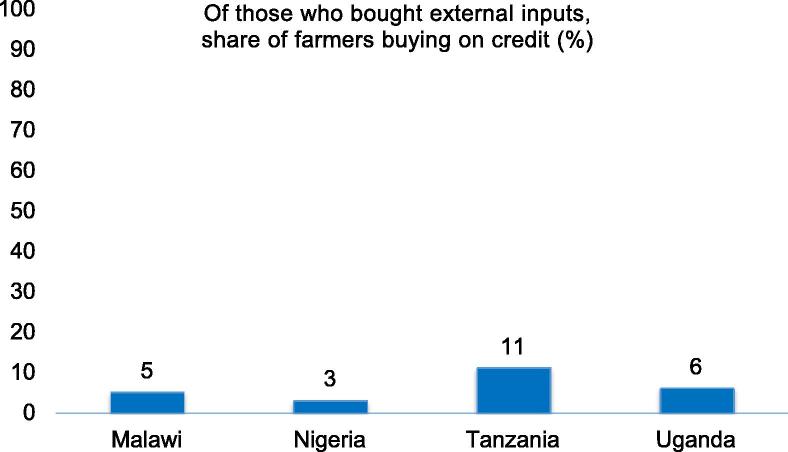
Virtually all purchases of modern inputs are financed from non-credit sources.

Source: Liverpool-Tasie et al. (2017).

***Farmers rarely use credit when purchasing farm inputs****:* The role of credit in rural transformation is well understood, but do African farmers make use of credit when purchasing modern inputs? Adjognon et al. (2017) show that credit use for fertilizer, pesticide or seed purchases is extremely low, across credit type (formal, informal, tied), crop (food or cash crop) and countries (Fig. 5). They estimate that on average only 6 percent of farmers use any form of credit to buy these inputs—at least in the four countries they cover (Malawi, Nigeria, Tanzania and Uganda). Larger farms are more likely to use credit, but interestingly, even there, the use of informal credit is found to be rare. Modern inputs are primarily financed through cash from nonfarm activities and crop sales instead.

While it is well documented that formal bank credit is seldom available to African farmers for input purchases, the working hypothesis is that farmers use tied credit with output and input traders and other sources of informal credit to finance the purchase of external inputs, while processors front inputs or cash for inputs in case of contract farming and cash cropping. The findings presented here contradict this, pointing to the important role of off-farm income and crop sales instead. While this should not be taken as proof of credit constraint as such, it does highlight the importance of the nonfarm sector for agricultural modernization and the intimate links between agriculture and off-farm employment, to which we return below. Broader (nonagricultural specific) rural development investments and policies will benefit agricultural development through different channels. In fact, the most common purpose of credit to a farming family in Africa is to finance the start-up costs of non-farm enterprises (or to finance consumption). This may be partially to help finance input purchases and increase agricultural productivity, an area for further investigation.

**Fig. 6:**
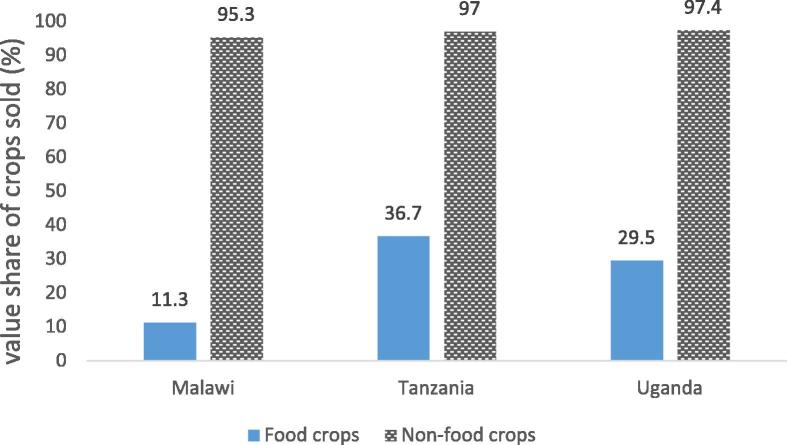
Food crop commercialization remains limited.

Source: Carletto et al. (2017).

**Market participation is widespread, but the extent of agricultural commercialization remains limited, without clear benefits for nutritional outcomes.** Taking the share of the gross value of crop sales to the gross value of total agricultural production, i.e. the crop commercialization index, as their measure of market participation or agricultural commercialization, Carletto et al. (2017) find that farmers sell on average around 20–25 percent of their crop output (a bit less in Malawi, slightly more in Uganda and Tanzania). Conditional on sales the rates amount to 20, 40 and 33 percent in Malawi, Uganda, and Tanzania respectively, indicating that while most farmers sell some crops in these three countries, the marketed shares remain limited. Conditional on planting and selling, 11, 37 and 30 percent of the value of food crops is commercialized in Malawi, Tanzania and Uganda respectively (Fig. 6). Unsurprisingly, commercialization rates rise with harvest size, but they are not confined to the traditional cash crops (which are fully commercialized). And even though they are less likely to sell, when they sell, female farmers tend to sell larger shares.

Using household and individual panel data, the authors further explore the relationship between agricultural commercialization, welfare and nutritional outcomes. Conventional wisdom suggests that the more farmers commercialize their operations through increased product-market orientation, the better off they can become. Greater market-orientation of agriculture would therefore be expected to raise incomes, improve consumption, and enhance nutritional outcomes in rural households (Braun and Kennedy, 1994).

The authors find little evidence of this in the three countries studied, underscoring that many factors other than the degree of agricultural commercialization intervene in shaping nutritional outcomes, including other agriculture related factors. The articles in the 2015 Journal of Development Studies Special Issue Vol 51, Issue 8 guest-edited by Carletto et al. (2015c), explore some of them, such as the role of diversity in crop production and livestock ownership. Livestock seems to emerge as particularly important and positively linked to nutrition, with crop production and diversity of production positively associated in certain contexts. Bio-fortification also pays off. Given the current emphasis on nutrition-sensitive agriculture, a better understanding of the transmission channels between crop choice, agricultural market engagement and nutritional outcomes continues to be a research priority.

***There is substantial excess seasonality in food prices.*** Although it is commonly accepted that seasonality permeates African livelihoods, surprisingly little attention is paid to it. Because of the seasonal nature of agricultural production, one area where seasonality manifests itself, is in food prices. But there is also very little systematic evidence on the extent of food price seasonality, and what is available, is largely dated. Better integration of domestic food markets today may explain part of the neglect. Gilbert et al. (2017) conclude that “while we all know about seasonality, it is very unclear precisely what it is we know.”

**Fig. 7:**
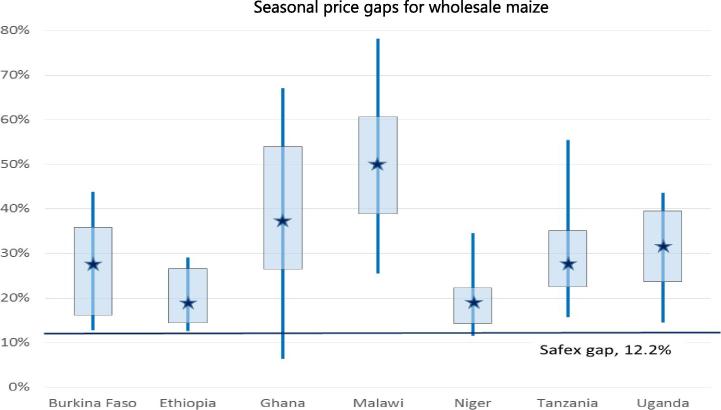
Excess seasonality in maize prices remains substantial and widespread. **Note**: seasonal price gaps, the percent mark-up of the peak over the trough month price, are calculated for each maize market in each country; stars indicate the median seasonal price gap across the markets examined in that country, the box borders indicate the 80th and 20th percentile and the endpoints of the vertical line the maximum and minimum. The SAFEX gap is the seasonal price gap for white maize observed in the South African Future Exchange market, which represents the international reference market.

Source: Gilbert et al. (2017).

The authors show that seasonality in staple crop prices can be substantial. They do so using trigonometric and sawtooth models to overcome some of the systematic upward bias in seasonal gap estimates from the common monthly dummy variable approach. This arises especially in shorter samples of 10–15 years, a phenomenon which has so far gone unnoticed.^13^ Maize prices in the 193 markets from the seven African countries studied are on average 33 percent higher during the peak months than during the troughs (Fig. 7). For rice the price gap is on average 16.5. These seasonal mark ups are two and a half to three times larger than in the international reference markets. Seasonality varies substantially across markets, but in virtually none of them is it lower than in the reference markets. Seasonality does not explain much of overall price volatility over the year.

The findings confirm the existence of substantial excess seasonality, for which there may be a host of reasons, including poor post-harvest handling, lack of storage facilities, lack of market integration as well as lack of coping capacity (possibly because of financial market failure) inducing sell-low, buy-back-high behavior (Stephens and Barrett, 2011). Follow-up analysis in Tanzania (Kaminski et al., 2016) further shows that the estimated food price seasonality also translates into seasonal variation in caloric intake of about 10 percent among poor urban households and rural net food sellers. Together the findings suggest that the current academic and policy neglect of price seasonality is premature.

### 3.3 Faltering structural transformation

Following the end of the commodity supercycle and the collapse in commodity prices since 2012, attention has shifted to structural transformation as the driver for growth and poverty reduction in Africa (Barrett et al., 2017). Given generally perceived lower labor productivity in agriculture and under-diversification of rural incomes, much is vested in accelerating the transition out of agriculture (Gollin et al., 2014). The extent to which (rural) household non-farm enterprises can be part of the solution remains unclear. Their productivity is generally considered low, but little systematic evidence is available.

The last three papers in the special issue revisit the evidence on the agricultural labor productivity gap (McCullough, 2017), document the recent patterns of rural employment and income diversification (Davis et al., 2017), and explore the prevalence, patterns and performance of rural nonfarm household enterprises (Nagler and Naudé, 2017). Together they provide key micro stylized facts to inform policy directions for the structural transformation of rural Africa.

***The agricultural labor productivity gap is smaller than commonly assumed and mainly due to underemployment, not intrinsic lower productivity in agriculture***. One common view, especially popular among macro economists, is that labor is intrinsically far less productive in agriculture than elsewhere in the economy, and that a great deal is thus to be gained from transferring labor out of agriculture, i.e. from accelerating the structural transformation. This view finds support in the national accounts which show that, in Africa, value added per nonagricultural worker is six times larger than the value added per agricultural worker. In developing countries, the ratio is 3.5.

Yet, this does not account for differences in human capital and income diversification across sectors. Recent work by Gollin et al. (2014) shows that adjusting for those factors would bring the ratio down to 3.3 for Africa (and 2.2 for developing countries). But even this may be misleading. In addition to neglecting differences in capital use across sectors, these macro numbers do not account very well for production for own consumption, which in developing countries makes up a substantial share of agricultural output as shown by the agricultural commercialization figures above. Using output measures from micro data instead, reduces the gap by another 20 percent in the ten countries for which Gollin et al. (2014) had data.

The paper by McCullough (2017) in this special issue takes the argument one step further still. Instead of comparing (micro based) output estimates *per worker*, she also uses detailed micro data on hours worked in both primary and secondary occupations to measure and compare labor productivity *per hour worked*. Doing so shrinks the labor productivity gap to 1.6 on average, across the four countries she studies, compared with 3.9 when using output per worker (Table 4).^14^ This is because workers in agriculture work fewer hours (700 hours per worker per year on average in the four countries she studies) than those outside agriculture (1850 hours). Not only is the agricultural labor productivity gap not as large as commonly portrayed, it follows mainly from underemployment and not from intrinsic lower productivity in agriculture.

This shifts the policy focus from getting people out of agriculture per se to making better use of labor in agriculture. In agriculture, work hours are constrained or rationed. This is possibly because of the seasonal nature of crop production, and relatedly, the seasonal availability of agricultural labor, another dimension of seasonality which deserves further investigation. In such a case, smallholder labor productivity could be raised by making more use of their labor off season, either on or off the farm. In environments with favorable temperature, water availability, and product demand, this can be done on the farm through the promotion of irrigation and horticulture enabling multiple crops a year, or through diversification into livestock products such as poultry, eggs or dairy, which are less seasonal.

Where demand for (seasonal) off-farm labor exists, the focus may be on reducing barriers to mobility, as even small initial travel costs (compared with the potential gains) may compound the effects of inexperience, uncertainty and credit constraints to prevent subsistence farmers from accessing it, as illustrated by Bryan et al. (2014) in Bangladesh.^15^ Or, in the absence of such labor demand, the role for off-farm employment generation nearby and thus secondary town development, becomes important in accelerating poverty reduction, as discussed by Ingelaere et al. (2017) in Tanzania.

***African households are not unduly tied to agriculture****:* The common view is that families in rural Africa rely more on agriculture compared with other parts of the developing world. Davis et al. (2017) revisit this using comparable employment and income aggregates from 41 national household surveys (14 from SSA of which 6 are LSMS-ISA) from 22 countries (9 from SSA). They confirm that agriculture remains the mainstay of rural livelihoods in SSA. Virtually all rural households have an on-farm activity (92 percent on average across countries). But this also holds in other regions (85 percent), with little change observed across countries over time or by GDP.

**Fig. 8:**
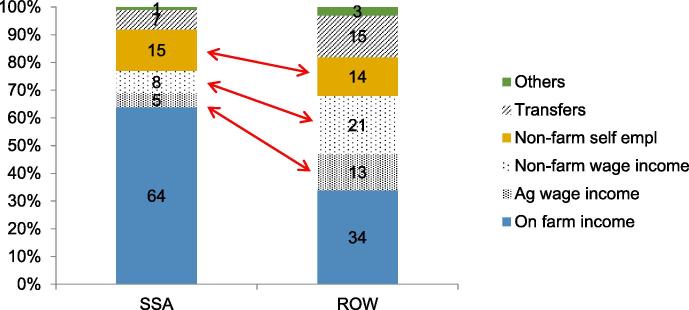
Rural Africa derives a much larger share of income from agriculture, a similar share from non-farm self-employment, but less from wages (in and outside agriculture). **Note**: ROW = Rest of the World.

Source: Davis et al. (2017).

Rural households also derive about two thirds of their income from on-farm agriculture, compared with one third (on average) in other developing countries (Fig. 8). But when differences in the level of development are taken into account (as reflected in GDP per capita), Africa is not on a different structural trajectory from the other developing regions. There are nonetheless some important differences.

Rural households in Africa are less engaged in wage employment, both on and off the farm. With the exception of Malawi, where it contributes 15 percent of income, the share of agricultural wage income is only five percent on average. This suggests that the second round effects from a food price increase through the agricultural wage labor market (Ivanic and Martin, 2008) are likely limited in SSA, unlike in India (Jacoby, 2016) or Bangladesh (Ravallion, 1990) where about a third of rural households are involved in agricultural wage labor, contributing 15–20 percent of income (Davis et al., 2017, Table A2–3). Far fewer households are also involved in nonfarm wage employment, even after controlling for the level of development, resulting in a small share of nonfarm wage income in total income (8 percent compared with 21 percent in the rest of the world). Most off-farm income in Africa is derived from informal self-employment.

Of course there are differences across African countries, and within countries, partly driven by agro-ecological potential and market access, which are discussed in more detail by the authors. Yet the overall employment and income patterns discerned here correspond to the picture emerging from the macro-data. There has been structural transformation away from agriculture in SSA over the past two decades—the agricultural share in GDP fell from 23 percent in 1995 to 17 percent in 2013, and the share of agricultural employment likewise fell by an estimated 10 percentage points (Barrett et al., 2017). Yet, it has been towards self-employment in (non-tradable) services, not wage employment in tradable manufacturing (Rodrik, 2016). This is partly because of Africa’s commodity boom, which fueled the emergence of consumption cities, characterized by higher shares of imports and employment in non-tradable services (Gollin et al., 2016). Given their prevalence, this raises the question of whether informal rural household enterprises can serve as pathways out of poverty.

***Households in rural Africa diversify into non-farm activities mainly for survival*.** While the dominant form of off-farm income, there is little systematic evidence on the prevalence, patterns and performance of Africa’s rural non-farm enterprises. Nagler and Naudé (2017) are the first to exploit the enterprise modules of the LSMS-ISA survey to characterize systematically the rural household enterprise landscape in SSA. They find that non-farm self-employment activities in the African household are indeed mostly oriented around survival. The evidence lies in the nature of these activities: most are small, unproductive, informal household enterprises, operated from home, without any non-household employees and often operating only for a portion of the year. They are concentrated in easy-to-enter activities, such as sales and trade, rather than in activities that require higher starting costs, such as transport services, or educational investment, such as professional services.

**Fig. 9:**
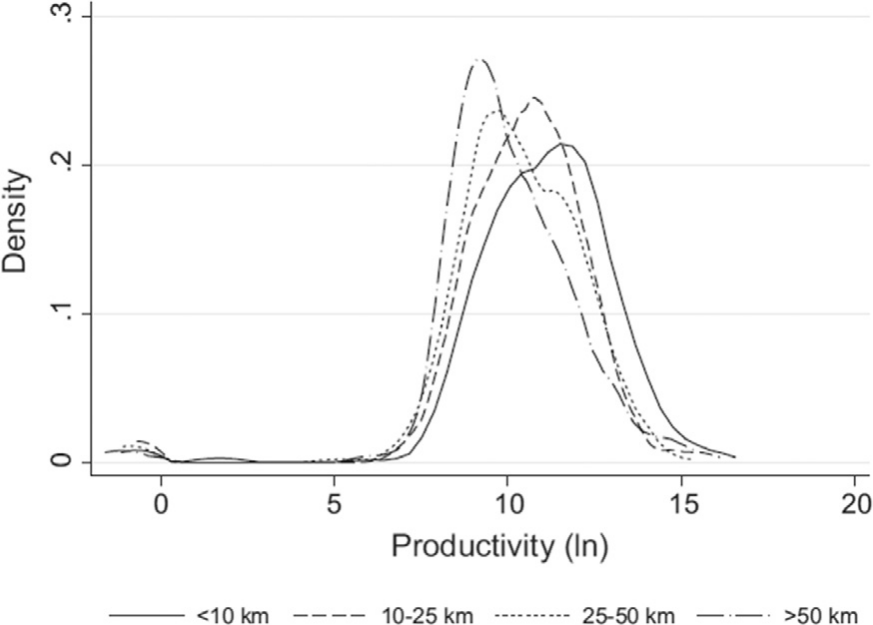
Enterprise productivity (Uganda) declines with distance from the urban center.

Source: Nagler and Naudé (2017).

But obviously not all are just there for survival, and labor productivity differs widely. Especially rural and female-headed enterprises, those located further away from urban centers (Fig. 9), and businesses that operate intermittently display lower labor productivity compared with urban and male-owned enterprises, or enterprises that operate throughout the year. Rural enterprises exit the market primarily because of a lack of profitability or finance, and due to idiosyncratic shocks. Nonetheless, the authors also show that when the conditions are right, households can seize the opportunities for enhancing family income. When households are better educated and have access to credit, they engage in agribusiness and trade throughout the year—not just in survival mode. The policy challenge is to create a business climate to foster such activities, which remains a tall order.

## 4 Concluding remarks

Stylized facts drive much of our research and policies, but robust stylized facts remain hard to come by, because of conceptual challenges, lack of data or insufficient incentives. All three apply when it comes to statistics on African agriculture and its farmers’ livelihoods. The papers in this special issue have exploited the newly available nationally representative LSMS-ISA survey data to begin addressing this void. They confront some of the more salient conventional wisdoms on agricultural technology, markets and farmers’ livelihoods with these data.

The findings reveal how a few of our stylized facts on African agriculture, and the policies they motivate, are wrong headed (agriculture’s large labor productivity gap and women’s disproportional labor contribution to agriculture). Many others call for a revision (the link between agricultural commercialization and nutrition, the assumption of input profitability) or fine-tuning (the missingness of factor markets and the extent of input use, land market operations and income diversification). They also call attention to neglected topics (seasonality) and open new lines of inquiry and policy attention (the role of agro-chemicals, the lack of agronomic knowledge, the reasons behind agricultural underemployment). And some simply stand up to the data, as one would hope (the survival orientation of most nonagricultural rural household enterprises, and the virtual absence of credit use for input acquisition).

Methodologically, the findings underscore the power of nationally representative data and cross-country standardization and comparison. They also highlight the power of data innovation and disaggregation at scale, as well as the power of descriptive statistics in shaping research and policy narratives. The hope is that the findings presented here catalyze further endeavors—to revisit our stylized facts in other areas and to update and deepen them as new data come along. Evidence-based policy-making requires sound facts as well as sound inference. With either one of them missing, researchers and policymakers alike risk flying blind.

## Figures and Tables

**Table 1:** Conventional wisdom about African agriculture: true or false?

Paper	The myth--what is the issue?	Myth or fact
*I. Production and technology adoption*		
1	African farmers use very low levels of modern inputs	Not generally true
2	Population growth and market access determine intensification	Not generally true
3	Given its profitability, fertilizer use is too low	Not true in Nigeria
4	Women provide the bulk of labor in African agriculture	False

*II. Market engagement*		
5	Factor markets are largely incomplete in Africa	True
6	Land markets play a minor role in African development	Increasingly false
7	Modern inputs are not financed through formal credit	True
8	Agricultural commercialization enhances nutrition	False
9	Seasonality in African food markets is fading	False

*III. Structural transformation*		
10	Labor is much less productive in agriculture	False
11	Incomes among African farmers are under-diversified	Largely false
12	Household non-farm enterprises only exist for survival	Largely true

**Table 2:** Fertilizer use and fertilizer use expansion do not pay on about half of the maize plots in the cereal-root cropping system in Nigeria.

Year	Full acquisition cost	Fertilizer available in the village
*Maize plots (%) for which net benefit from fertilizer use is positive for a risk neutral farmer (AVCR ≥ 1)*		
2010	51	86
2012	56	88

*Maize plots (%) for which expanding fertilizer use is profit increasing for a risk neutral farmer (MVCR ≥ 1)*		
2010	49	70
2012	53	86

Source: Liverpool-Tasie et al. (2017).

**Table 3:** While generally incomplete, factor markets are not generally missing.

% agricultural households	Ethiopia	Malawi	Niger	Tanzania	Uganda	Average
Rent/borrow land	32.7	24.9	30.9	19.6	38.7	29.4
Hiring labor	30.2	40.1	48.7	30.1	45.2	38.9
Take loan/access credit	27.5	13.3	–	13.3	40.8	23.7

Source: Dillon and Barrett (2017).

**Table 4:** Underemployment explains most of the agricultural labor productivity gap.

Nonagricultural/agricultural output	Ethiopia	Malawi	Uganda	Tanzania	Average
Per person productivity gap	2.25	4.76	4.48	4.20	3.92
Employment gaps	2.66	3.30	2.10	2.22	2.57
Per-hour productivity gaps	0.85	1.44	2.13	1.90	1.58

Source: McCullough (2017).

## Footnotes

1 http://www.politifact.com/punditfact/article/2014/jul/03/meet-zombie-stat-just-wont-die/

2 See for example Rao (2007, Ch3) on measuring seasonality.

3 The Initiative is financed by a grant from the Bill and Melinda Gates Foundation, together with other contributors, and managed by the Development Economics Data Group of the World Bank Group. Several other data initiatives are also underway to remedy the agricultural data situation, such as the Global Strategy to Improve Agricultural and Rural Statistics, and the ensuing regional Action Plans.

4 Other participating institutions included the Alliance for a Green Revolution in Africa, Cornell University, the Food and Agriculture Organization, the London School of Economics, the Maastricht School of Management, the University of Pretoria, the University of Rome Tor Vergata, the University of Trento, and Yale University. For a detailed description of the project and its collaborators, see http://www.worldbank.org/en/programs/africa-myths-and-facts.

5 For a detailed description and access to the data and their documentation, see http://www.worldbank.org/lsms.

6 Ongoing research on other, related stylized facts, not included in this special issue, are for example the notion that the majority of rural households are net food buyers (Palacios-Lopez et al., 2017a), the notion that African youth is exiting agriculture en masse (Maiga et al., 2017) and the notion that droughts are the main hazard in African livelihoods (Nikoloski et al., 2017).

7 The latest official numbers for SSA (2013) put the average 17.5 kg/ha (World Bank, 2017).

8 See Tamru et al. (2016) for a recent analysis of the patterns, drivers, and labor productivity implications of the rapid expansion of herbicide use in smallholder cereal agriculture in Ethiopia over the past decade.

9 Comparing crop variety assessments by farmers with DNA fingerprints Ilukor et al. (2017) show that improved and hybrid variety cultivation is more widespread than commonly assumed by farmers in Uganda, even though the improved cultivated varieties are no longer pure.

10 It is commonly thought that modern inputs are seldom adopted in isolation since there are important complementarities between particular sets of inputs making it advantageous to use them together (Yilma and Berger, 2006, Nyangena and Juma, 2014, Abay et al., 2016).

11 Gollin et al. (2016) document how Africa’s commodity boom during the 2000s fueled economic growth in many resource rich countries as well as urbanization, in particular the emergence of consumption cities, characterized by higher shares of imports (including of food) and employment in non-tradable services, as opposed to tradable manufacturing or services.

12 Maize is one of the three most important cereals grown in Nigeria along with sorghum and millet. Maize plots in the cereal-root crop farming system represent about 60 percent of the plots in the study sample.

13 Unlike most of the studies reported here, the main data source for this study are not the LSMS-ISA household surveys, but rather price data obtained in key markets across the seven countries covered, five of which are LSMS-ISA countries.

14 These gap estimates are plausibly still upper bounds. They do not correct for cross-sectoral differences in human and physical capital.

15 They show how an US$ 8.5 grant or credit incentive (conditional on seasonal migration and equivalent to the round trip fare), induced 22 percent of households to send a seasonal migrant, increasing consumption at origin by 30–50 percent and re-migration 1–3 years after the program by 8–10 percentage points.
